# Ocular and musculoskeletal changes in the pediatric population using gadgets

**DOI:** 10.22336/rjo.2022.48

**Published:** 2022

**Authors:** Irina Andreea Pavel, Bogdan Savu, Cristina Petronela Chiriac, Camelia Margareta Bogdănici

**Affiliations:** * Discipline of Ophthalmology, “Grigore T. Popa” University of Medicine and Pharmacy, Iași, Romania; ** Discipline of Pediatrics, “Grigore T. Popa” University of Medicine and Pharmacy, Iași, Romania; *** Director of Health Care, “Sf. Spiridon” Emergency Hospital, Iași, Romania

**Keywords:** Computer Vision Syndrome, children, digital-screen electronic devices

## Abstract

**Objective.** Analysis of ophthalmological and musculoskeletal changes secondary to the use of electronic devices with digital screen, such as smartphones, laptops, computers or tablets.

**Material and Methods.** This paper represents is a prospective observational study of 35 participants with ages between 6 and 17. The ophthalmological exam was carried out for all participants in the Ophthalmological Clinic of “Sf. Spiridon” Emergency Hospital, Iaşi, and the exam of musculoskeletal disorders took place at “Sf. Maria” Pediatrics Hospital, Iaşi. A questionnaire including 14 questions was also distributed for the symptoms caused by the use of digital screen electronic devices.

**Results.** The mean age of participants was 11,29 ± 3,54 years, predominantly female children (62,9%). Convergence insufficiency has been identified in all patients with accommodative disorders and in 18.2% of the children with amblyopia (p = 0.001). The frequency of cases with dry eye syndrome (DES) was 9.1% in the patients with accommodative disorders and 18.2% in the patients with amblyopia. In the entire studied group of patients, the smartphone was the most frequently used electronic device, being found in 77.1% of the cases. As for the gender, about 54% of the boys spent more than 5 hours on electronic devices, while 54.5% of the girls spent between 3 to 5 hours. Among the symptoms that occur during the use of gadgets, pain in the neck, shoulders and back was found most often, being identified in 29 participants.

**Conclusion.** Pre-existent ophthalmological symptoms can be exacerbated by prolonged use of digital screen electronic devices. Musculoskeletal symptoms were encountered in high numbers in all participants, which suggests that musculoskeletal changes must be treated with great importance in Computer Vision Syndrome. Also, the symptomatology determined by the use of gadgets was more frequently associated with males than females.

**Abbreviations:** CVS = computer vision syndrome, VA = visual acuity, VDT= visual display terminals, DES = dry eye syndrome

## Introduction

In a world that is constantly changing and evolving, more and more parents are concluding that the use of the computer will lead to the well-being of children by stimulating creativity, means of expression and enriching knowledge. On one hand, the computer era has brought many benefits, but on the other hand, it has also determined the overload of the human body. Prolonged and repeated exposure to digital screens causes a lot of ophthalmological and systemic problems.

Computer Vision Syndrome (CVS) represents a series of ophthalmological, musculoskeletal, and behavioral disorders caused by the prolonged use of visual display terminals (VDT). The first symptoms that appear are headaches, stinging and redness of the eyes, blurred vision, pain in the muscles of the neck and back, eye fatigue, visual accommodation difficulties and attention disorders [**1**]. Computer Vision Syndrome has been a recognized health problem for over 20 years and, given the significant growth in digital device usage in the last years, millions of individuals of all ages are at risk of CVS [**2**]. The usage of electronic visual display devices is no longer limited to desktop computers located in the workplace. Digital electronic screens such as laptops, smartphones or tablets are used nowadays in the workplace, at home or in any other location [**3**]. Recent studies found that the prevalence of asthenopia among the VDT users is between 55% and 81% [**4**].

The aim of this study was to asses ophthalmic and systemic changes secondary to the use of digital-screen electronic devices such as laptops, tablets, smartphones or computers. 

## Material and methods

We conducted a prospective, observational, case-control study, which included 35 patients divided into three groups:

• a group of 11 children with accommodative disorders;

• a group with the same number of subjects that included children with amblyopia;

• the control group consisting of 13 subjects who did not have ophthalmic diseases.

The patients were selected from the Ophthalmology Clinic of “Sf. Spiridon” Emergency Hospital, Iași, between February 2021 and November 2021.

The ophthalmological examination included the following: visual acuity (VA) measurement at the Snellen chart, refraction measurement with the help of the auto kerato-refractometer, anterior segment examination at the slit lamp, Schirmer test and the orthoptic examination with the help of the synoptophore that included the measurement of fusional amplitude, as well as the examination of binocular vision. 

The consultation of the musculoskeletal system was performed at “Sf. Maria” Children’s Hospital, Iași, by a pediatric orthopedist, which also included a chest X-ray.

Data were collected through clinical examination of patients, as well as using a questionnaire that included 14 questions regarding the symptoms of the gadget use. The questionnaires were completed by the children together with their parents.

Each patient and the patient’s legal representative signed the “Informed consent” certifying the agreement to participate in the study, to take part in the examinations related to it, as well as to consent to the publication of the results for scientific purposes and under the full protection of anonymity. Prior to enrolling patients, the study protocol was approved by the Ethics Committee of “Grigore T. Popa” University of Medicine and Pharmacy Iași.

After enrolling patients, all parameters used in the study were recorded in a standardized database (Microsoft Excel 2013), appropriately coded. The data were systematized and centralized in an SPSS 18.0 database and were processed with the statistical functions for which they were suitable. 

## Results

The patients included in the study were aged between 6 and 17 years, the group average being 11.29 ± 3.54 years. After applying the ANOVA test (p = 0.539), the post-hoc Bonferroni correction test was performed, which showed that the mean age in the control group was slightly lower compared to that recorded in patients with accommodative disorders (10.54 vs. 11.27 years; p = 0.999) or in those with amblyopia (10.54 vs. 12.18 years; p = 0.811).

**Fig. 1:**
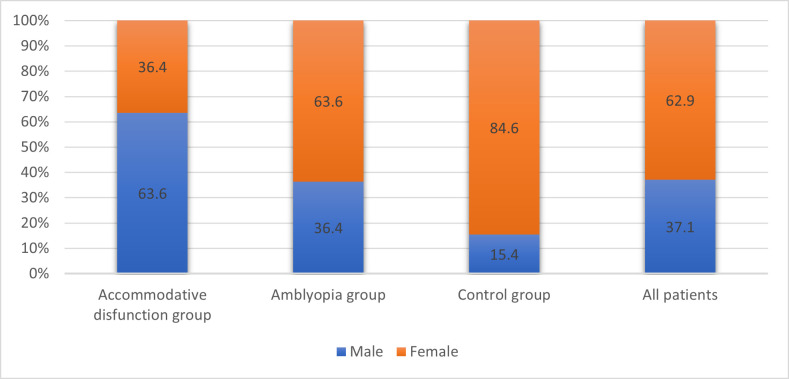
Groups structure depending on gender

In our study, female children predominated (62.9% vs. 37.1%), the ratio of quotas being 2/ 1, but it should be noted that in the case of patients with accommodative disorders, male children predominated (63.6%), while in the case of patients with amblyopia (63.6%) and in the control group (84.6%) female gender predominated (p = 0.046) (**Fig. 1**).

The series of values for VA with and without correction of the right eye or left eye were homogeneous in all groups analyzed, the mean values were close to the median values, and the results of the Skewness test were in the range [-2 ÷ +2]. After applying the ANOVA test that identifies significant differences in mean values between study groups (p = 0.001), the post-hoc Bonferroni correction test was performed, which showed that the mean VA with and without correction in the group of patients with amblyopia was significantly lower compared to the group of patients with accommodative dysfunction (p = 0.001) and the control group (p = 0.001), both in the right eye and in the left eye (**Table 1**, **2**).

**Table 1:** Descriptive data of VA without correction compared on study groups

			Right eye			Left eye	
		Accommodative dysfunction group	Amblyopia group	Control group	Accommodative dysfunction group	Amblyopia group	Control group
N		11	11	13	11	11	13
Mean		0,72	0,40 a)	1,00 b) a)	0,73	0,40 a)	1,00 b) a)
Median		0,80	0,40	1	0,80	0,40	1
Standard Deviation		0,31	0,13	0	0,31	0,11	0
Variance		0,10	0,02	0	0,09	0,01	0
Skewness Test		-0,678	-0,304		-0,619	0,558	
Er. standard Skewness		0,661	0,661	0,616	0,661	0,661	0,661
Minimum		0,16	0,20	1	0,20	0,30	1
Maximum		1,00	0,60	1	1,00	0,60	1
Percentile	25	0,50	0,30	1	0,50	0,30	1
	50	0,80	0,40	1	0,80	0,40	1
	75	1,00	0,50	1	1,00	0,50	1
a) p<0,001 b) p<0,01						

**Table 2:** Descriptive data of VA with correction compared on study groups

			Right eye			Left eye	
		Accommodative dysfunction group	Amblyopia group	Control group	Accommodative dysfunction group	Amblyopia group	Control group
N		11	11	11	11	11	13
Mean		1	0,77 a)	1,00 ns) a)	1	0,75 a)	1 ns) a)
Median		1	0,80	1	1	0,80	1
Standard Deviation		0	0,06	0	0	0,08	0
Variance		0	0,04	0	0	0,01	0
Skewness Test			0,291			-1,505	
Er. standard Skewness		0,661	0,661	0,616	0,661	0,661	0,616
Minimum		1	0,70	1	1	0,60	1
Maximum		1	0,90	1	1	0,80	1
Percentile	25	1	0,70	1	1	0,70	1
	50	1	0,80	1	1	0,80	1
	75	1	0,80	1	1	0,80	1

**Fig. 2:**
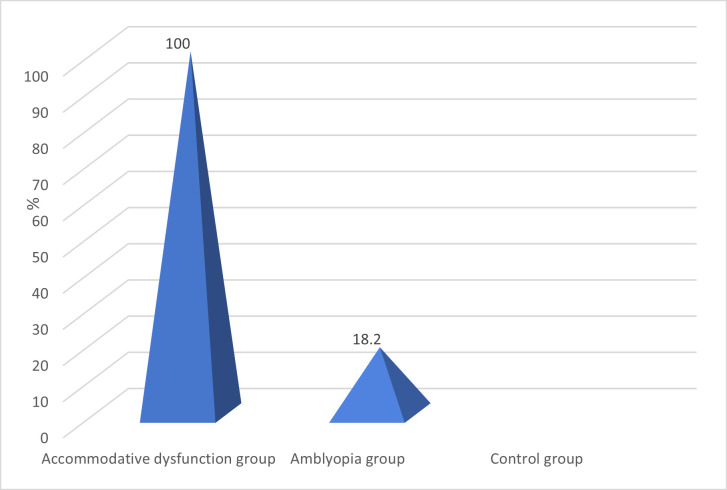
Distribution of cases with convergence insufficiency in all study groups

Approximately 1/ 2 of the children with low myopia were from the groups with accommodative disorders and amblyopia (p = 0.001). Moderate myopia was found in 9.1% of the children with amblyopia (p = 0.304), and moderate hyperopia was found in 27.3% of the patients with amblyopia (p = 0.023). Convergence insufficiency was identified in all children with accommodative dysfunctions and in 18.2% of the patients in the amblyopia group (p = 0.001) (**Fig. 2**).

The frequency of cases with scoliosis was 45.5% in both groups with accommodative disorders and amblyopia and 38.5% in the control group (p = 0.921). Scoliosis was associated with myopia in 4 patients from both accommodative dysfunctions group and amblyopia group. Moreover, scoliosis was associated with convergence insufficiency in 5 children.

The frequency of cases with dry eye syndrome (DES) was 9.1% in the group with accommodative disorders and 18.2% in the group with amblyopia. The DES was diagnosed with Schirmer I test, abnormal values being considered less than 10 mm of moisture on the filter paper in 5 minutes. The comparatively associated pathologies in the study groups are presented in **Table 3**.

**Table 3:** Comparatively associated pathologies on study groups

Associated pathology	Accommodative disorders group (n=11)	Amblyopia group (n=11)	Chi2 test
			p
Myopia	6 (54,5%)	10 (90,9%)	0,061
Myopia + scoliosis	4 (36,4%)	4 (36,4%)	1,000
Convergence insufficiency	11 (100%)	2 (18,2%)	0,001
Convergence insufficiency + scoliosis	5 (45,5%)	0 (0%)	0,013
DES	1 (9,1%)	2 (18,2%)	0,544
DES + myopia	1 (9,1%)	2 (18,2%)	0,544
DES + convergence insufficiency	1 (9,1%)	0 (0%)	0,317
DES + scoliosis	1 (9,1%)	1 (9,1%)	1,000

In the entire studied group of patients, the smartphone was the most frequently used electronic device, being found in 77.1% of the cases and the laptop was the second one.

**Fig. 3:**
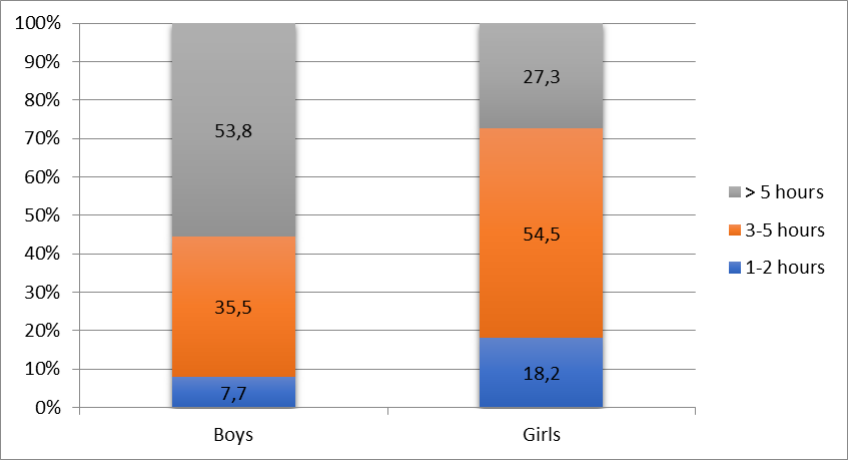
Correlation between gender and daily time spent on gadgets

Regarding gender, about 54% of the boys spent more than 5 hours on electronic devices, while 54.5% of the girls spent between 3 to 5 hours (p = 0.268) (**Fig. 3**).

Children who spent more than 5 hours a day on electronic devices had an average age of 12.92 ± 2.69 years, while those who spent 1-2 hours a day had an average age of 6.20 ± 0.45 years (p = 0.001).

**Fig. 4:**
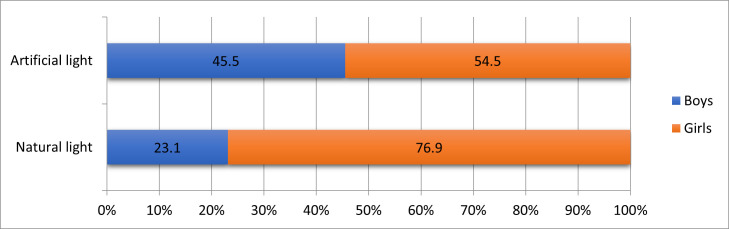
Frequency of the most commonly used type of lighting by gender

 The preferred type of lighting was artificial light (52.9% of the total cases). In our study, 54.5% of those who preferred artificial light and 76.9% of those who preferred natural light were girls (p = 0.178) (**Fig. 4**).

Out of a total of 17 patients wearing glasses, 11 were in the amblyopia group and 6 in the accommodative disorder group. Of these, diopters have changed in 68.8% in the last 12 months. From the total of 17 children (48.6%) who wore glasses, 58.9% were girls.

In all patients, the most common symptoms associated with the use of electronic devices were neck, shoulder and back pain, being found in 82.9% of the cases (29 patients). Of these, 10 children were from the group with amblyopia, 10 from the group with accommodative disorders and 9 from the control group.

Eye fatigue was the second most common symptom associated with the use of gadgets, found in 57.1% of cases (20 patients), being observed in all patients with convergence insufficiency (13 cases).

Irritated red eyes were found in 54.3% of subjects (19 patients), 8 from the group with amblyopia, 7 from the group with accommodative disorders and 4 from the control group.

Dry eye was described by 51.4% of patients (18 cases), 9 in the group with accommodative disorders, 7 in the group with amblyopia and 2 in the control group.

Blurred vision occurred in 48.6% of patients (17 cases), 11 cases in the group with accommodative disorders, 5 in the group with amblyopia and 1 in the control group.

In males versus females, the most common symptoms associated with gadgets use were neck, shoulder, and back pain (84.6% vs. 81.8%; p = 0.831), blurred vision (53.8% vs. 45.5%; p = 0.631), headache (38.5% vs. 31.8%; p = 0.690), diplopia (23.1% vs. 18.2%; p = 0.728) and tearing (15,4% vs. 4.5%; p = 0.278) (**Table 4**).

**Table 4:** Frequency of symptoms associated with gadgets use by gender

Symptoms	Boys	Girls	Chi2 test (p)	OR	IC95%
Shoulder, neck and back pain	11 (84,6%)	18 (81,8%)	0,831	1,22	0,19-7,82
Headache	5 (38,5%)	7 (31,8%)	0,690	1,34	0,32-5,61
Blurry vision	7 (53,8%)	10 (45,5%)	0,631	1,40	0,35-5,54
Eye fatigue	6 (46,2%)	14 (63,6%)	0,313	0,49	0,12-1,98
Dry eye	6 (46,2%)	12 (54,5%)	0,631	0,71	0,18-2,83
Diplopia	3 (23,1%)	4 (18,2%)	0,728	1,35	0,25-7,28
Tearing	2 (15,4%)	1 (4,5%)	0,278	3,82	0,31-46,9

**Fig. 5:**
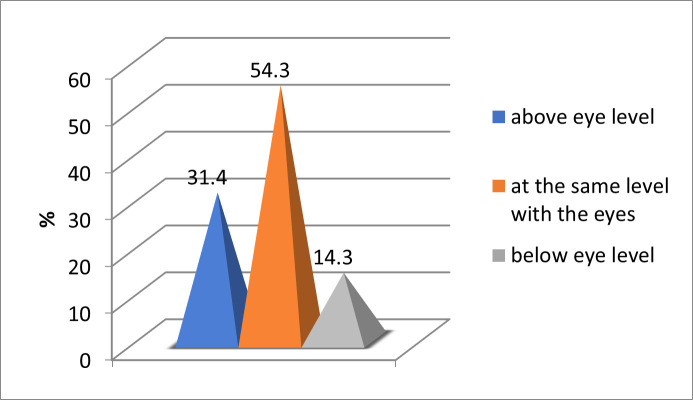
Laptop/ computer screen positioning frequency

Most frequent, the center of the computer/ laptop screen was positioned at the same level with the eyes (54.3%) (**Fig. 5**).

The values for the distance between the screen and the eyes were homogeneous, suggesting that tests of statistical significance variations in the range of 30-50 cm could be applied. Therefore, the group average was 36.29 cm ± 5.19, median 35 cm and Skewness test result p = 0.0448 (**Table 5**).

**Table 5:** Descriptive data regarding screen distance (cm)

		Total patients
N		35
Mean		36,29
Median		35
Standard Deviation		5,19
Variance		26,98
Skewness Test		0,048
Er. standard Skewness		0,398
Minimum		30
Maximum		50
Percentile	25	30
	50	35
	75	40

## Discussions

The technology revolution has contributed to a dramatic increase in the number of Internet users globally, from 147 million (4% of the world’s population) in 1998 to over 4 billion (53% of the world’s population) in 2018 [**5**].

Globally, smartphone usage increased from 21.6% in 2014 to 34.7% in 2018. Moreover, children are starting to use gadgets from a very young age, so 2-year-olds use these devices for about 2 hours a day [**6**].

A study conducted on children with ages between 9 and 11 years in 12 countries found that 54.2% of them exceeded the maximum daily use time (approximately 2 hours) of digital screen electronic devices [**7**].

Another recent study from China, conducted on a group of 19,934 students in grades IV-V, showed an association between prolonged use of computers and smartphones and significant refractive errors, in contrast to watching TV and studying for school subjects, which did not show any association [**8**].

A recent study by De-Hita-Cantalejo et al. showed that accommodative insufficiency is often associated with severe symptoms of CVS [**9**]. These results are similar with data from our study, showing that all patients with convergence insufficiency had eye fatigue and blurred vision when using gadgets.

The actual viewing distance and gaze angle were in accordance with the arranging of the working place, the height of the material being observed together with the physical size of the individual.

The United States Occupational Safety and Health Administration (OSHA) claimed that the ideal viewing distance for a VDT is among 50 and 100 cm. In addition, they suggested that the center of the VDT should be situated 15-20° under the horizontal eye level and the whole visual area of the screen should be situated in a manner that the downward viewing angle is never higher than 60°.

It should also be mentioned that laptops and computers are usually used in downward gaze while smartphones can be used in primary or downward gaze [**3**].

In a study conducted by Ranasinghe and col., the mean angle of gaze to the monitor was higher in those with CVS (31.9° ± 14.5°) than in those without CVS (29.9° ± 14.8°) [**10**]. Another study from 2021 showed that one of the most common risk factors associated with CVS was the improper gaze angle as the screen edge was situated at or above the horizontal eye level, which was recorded in 28.2% of patients [**11**]. In our study, the computer/ laptop screen was positioned most often at the same level with the gaze (54.3%), above the gaze level (31.4%) and below the gaze level (14.3%). These data suggested that 85.7% of all patients in our study did not look correctly at the computer/ laptop screen, which is a major risk factor for CVS.

A recent study found that a screen distance less than 40 cm is significantly associated with eye fatigue [**12**]. In our study, the average screen distance was 36.29 cm ± 5.19, suggesting a much shorter distance from current recommendations (50-100 cm), which is another important risk factor for CVS.

The use of artificial light has also been shown to be significantly correlated with eye strain, redness, and tearing [**12**]. In the present study, the preferred type of lighting was artificial light (52.9%), which highlighted the association between ambient lighting and eye symptoms. 

Recent studies have shown that CVS symptoms were more commonly associated with males than females [**12**,**13**]. These data were similar to the results of our study, which showed that in males compared to females, the symptoms that occurred more frequently when using electronic devices were neck, shoulder and back pain (84.6% vs. 81.8%; p = 0.831), blurred vision (53.8% vs. 45.5%; p = 0.631), headaches (38.5% vs. 31.8%; p = 0.690), double vision (23.1% vs. 18.2%; p = 0.728) and tearing (15.4% vs. 4.5%; p = 0.278).

The association between VDT use and DED, especially smartphone use, has also been identified in school-age children. Among a group of schoolchildren in Korea, the prevalence of smartphone use was increased among scholars with DED (71.4%) than among scholars without DED. Moreover, the duration of smartphone usage per day and total duration of digital screen use per day were associated with a higher risk of DED. On the other hand, daily duration of computer or TV use was not found to be associated with DED [**14**]. In our study, DES was diagnosed with Schirmer I test for 9.1% of the patients in the group with accommodative disorders and 18.2% in the group with amblyopia. On the other hand, 51.4% of all patients included in the study declared that they felt dry eyes while they used gadgets. 

Even though the ocular problems are the most common encountered complaining among individuals with CVS, extraocular symptoms presented their own concerns, too. This prolonged time spent in front of VDT can produce extraocular symptoms such as headaches, sleep disorders and depression [**15**,**16**]. Musculoskeletal symptoms include neck, shoulder or back pain [**17**,**18**].

The continuous use of digital screen devices may induce abnormal forward bending posture of the neck that can affect the anatomical structures. It has been found that the neck flexion angle is higher in case of smartphones use and may influence the muscle fatigue and pain of the upper trapezius [**19**]. A study conducted by Turkistani et al. reported that shoulder and neck pain were the most frequent musculoskeletal symptoms, found in 50.2% of the participants. Moreover, back pain was found in 44.9% of the participants [**20**].

Other factors that contribute to back pain are improper placement of keyboards or screens and improper office design [**21**]. When the digital display is positioned too high or too low, it can cause back pain and incorrect posture at the office [**22**]. Uncomfortable furniture of the wrong size and shape can also cause back pain [**23**]. This data is consistent with the results of our study, which showed that in all patients, the symptoms that occur most frequently when using electronic devices were neck, shoulder and back pain, being found in 82.9% of the cases.

A study conducted in 2021 on children with ages between 10 and 14 years old, concluded that increased computer use is associated with trunk asymmetry [**24**]. In our study, we found that scoliosis was diagnosed in 45.5% of the patients in both groups with accommodative disorders and amblyopia and in 38.5% in the control group. These findings suggested that the prolonged use of gadgets negatively impacted the health-related quality of life in children and early adolescents.

The main strength of this study is represented by its complexity because an ophthalmological examination and a musculoskeletal examination that included an X-ray were performed for all the patients. Moreover, a questionnaire for the symptoms caused by the use of gadgets was also completed by all patients. Furthermore, our findings showed that musculoskeletal symptoms and scoliosis were found in many participants.

The main weakness is represented by the small study group. Because this research was conducted during the peak of the COVID-19 pandemics, patients were reluctant to come to the hospitals. Further studies need to be done to strengthen the evidence from our paper.

## Conclusions

Pre-existing ophthalmic symptoms in groups with amblyopia and accommodative disorders may be exacerbated by the use of gadgets. Moreover, the symptoms caused by CVS were more frequently associated with males than females. Attention should be paid to musculoskeletal symptoms, which were encountered in large numbers in all participants and scoliosis was diagnosed in all study groups for an important number of children. Finally, it is very important to inform children and their parents about the ambient lighting and the correct positioning of the screen. A significant number of our study participants did not know how to use gadgets correctly.

**Conflict of Interest statement**

The authors state no conflict of interest.

**Informed Consent and Human and Animal Rights statement**

An informed consent has been obtained from all individuals included in this study or from their legal representatives.

**Authorization for the use of human subjects**

Ethical approval: The research related to human use complies with all the relevant national regulations, institutional policies, is in accordance with the tenets of the Helsinki Declaration, and has been approved by the Ethics Committee of “Grigore T. Popa” University of Medicine and Pharmacy Iași.

**Acknowledgements**

None.

**Sources of Funding**

None.

**Disclosures**

None.
